# Service quality of private hospitals: The Iranian Patients' perspective

**DOI:** 10.1186/1472-6963-12-31

**Published:** 2012-02-02

**Authors:** Asghar Zarei, Mohammad Arab, Abbas Rahimi Froushani, Arash Rashidian, S Mahmoud Ghazi Tabatabaei

**Affiliations:** 1 Department of Health Management and Economics, School of Public Health, Tehran University of Medical Sciences, Tehran, Iran; 2 Department of Epidemiology and Biostatistics, School of Public Health, Tehran University of Medical Sciences, Tehran, Iran; 3 Department of Demography, School of Social Science, University of Tehran, Tehran, Iran

## Abstract

**Background:**

Highly competitive market in the private hospital industry has caused increasing pressure on them to provide services with higher quality. The aim of this study was to determine the different dimensions of the service quality in the private hospitals of Iran and evaluating the service quality from the patients' perspective.

**Methods:**

A cross-sectional study was conducted between October and November 2010 in Tehran, Iran. The study sample was composed of 983 patients randomly selected from 8 private general hospitals. The study questionnaire was the SERVQUAL questionnaire, consisting of 21 items in service quality dimensions.

**Results:**

The result of factor analysis revealed 3 factors, explaining 69% of the total variance. The total mean score of patients' expectation and perception was 4.91(SD = 0.2) and 4.02(SD = 0.6), respectively. The highest expectation and perception related to the tangibles dimension and the lowest expectation and perception related to the empathy dimension. The differences between perception and expectation were significant (p < 0.001). There was a significant difference between the expectations scores based on gender, education level, and previous hospitalization in that same hospital. Also, there was a significant difference between the perception scores based on insurance coverage, average length of stay, and patients' health conditions on discharge.

**Conclusion:**

The results showed that SERVQUAL is a valid, reliable, and flexible instrument to monitor and measure the quality of the services in private hospitals of Iran. Our findings clarified the importance of creating a strong relationship between patients and the hospital practitioners/personnel and the need for hospital staff to be responsive, credible, and empathetic when dealing with patients.

## Background

Service sector is the rapidly growing area of the world economy and the health services organizations play an important role in such growth [1,2]. During the recent decade, the number of private centers providing health care services in Iran has been ever increasingly growing, and the private health care services market has turned out to be a competitive environment. Based on the statistics issued by the Ministry of Health of Iran (2009), 54 (40%) of 134 private hospitals active in the health sector of Iran and possessing 48% of the hospital beds of this sector have been operating in Tehran, the capital of Islamic Republic of Iran [3]. Highly competitive market in the private hospital industry has caused increasing pressure on them to provide services with higher quality.

Quality is considered a key factor in differentiation and excellence of services and is a potential source of sustainable competitive advantage so that its understanding, measurement, and improvement are important challenges for all health services organizations [4,5]. Hospitals provide similar services with different quality. The quality can be used as a strategic differentiation for establishing a distinctive advantage, those difficult for rivals to follow or copy [6]. Many of researchers have emphasized on the importance of determining role of quality in hospital choice by the patients, as well as satisfying and retaining customers and have claimed that the improvement of the quality of hospital services will increase the number of satisfied patients and thereby customer loyalty [5,7].

Quality in health services entails two dimensions: technical quality (outcome quality) and functional quality (process quality). Technical quality focuses on the accuracy of medical diagnoses and procedures whereas functional quality refers to the way in which health care services are delivered to patients [8]. Because most of patients lack the required knowledge for evaluating the technical quality of the services, their evaluation of quality is based on the medical care process [9].

Providing patients with the services according to their needs and expectations is crucial for survival and success of the organization in the competitive environment of the health care market [10]. Accurate recognition of the customers' needs and expectations is the most important step in defining and delivering high-quality services [11]. The patients' expectations are derived from their perception of the ideal care standards or their previous experiences in the use of services [12]. Different study results show that meeting the patients' expectations is related to his/her high satisfaction from the related services, in the same way as unmet expectations relate to dissatisfaction [13]. After delivering the services, service providers also must monitor how well the customers' expectations have been met.

Different methods exist for determining the patients' expectations and the way they are met. However, the SERVQUAL model, developed by Parasuraman *et al *[14], is one of the best and most used models for evaluating customer expectations and their perceptions of the quality of the services. In this model, the quality is equal to performance minus expectations. SERVQUAL is based on the idea that the quality is a subjective evaluation of the customer, as the service is not a physical item but an experience. Hence, customer perception is better compared with other measures of performance [15]. SERVQUAL is useful in showing the difference between the patients' preferences and his/her actual experience and specifies the areas that need improvement. The analysis of service quality enables hospital management to allocating the financial resources for improving performance in the areas that have more influential on the customers' perception of service quality [6].

Studies carried out in Iran on the quality of the health services have been basically focused on the primary health care [16,17]. To the best of our knowledge, the present study is the first one to investigate the service quality of private hospitals in Iran. This study follows the aim of determining the different dimensions of the quality of the services being provided in private hospitals of Iran and evaluating service quality from the patients' perspective.

## Methods

A cross-sectional study was conducted between October and November 2010 in Tehran, the capital of Islamic Republic of Iran.

### Sampling

The study sample was selected from among all patients who were hospitalized in private hospitals of Tehran. Eight general hospitals were considered for investigation and the samples were divided among the 8 hospitals based on proportionality to the size. The inclusion criteria comprised adult patients aged 15 years and older who were stayed at least 24 hours in the hospital and willing to participate in the study. The samples were selected randomly in each hospital, and the questionnaires were given to them on the day of discharge. The aim of the study was explained to patients, and they were assured of the privacy of their information. The illiterate patients were interviewed by a trained interviewer. Finally, 983 of the 1100 questionnaires distributed between the patients (response rate = 89%) were filled out and gathered for analysis.

### Survey instrument

The study questionnaire was composed of 2 parts: the first part includes 8 questions relating to the socio-demographic data of the patient. In the second part, the SERVQUAL questionnaire [18], with some modifications that are suitable for hospital environment, was used for assessing the patients' expectations and perceptions of service quality. The questionnaire included 21 items in 5 service quality dimensions: tangibles (4 items), reliability (4 items), responsiveness (4 items), assurance (4 items), and empathy (5 items). The SERVQUAL questionnaire has been translated to an Iranian language, and the Farsi version was available.

### Analysis

SERVQUAL has been tested in health care environments and has produced various results (from 1 to 9 dimensions), and there is no consensus on the number of quality dimensions [19]. Hence, because this was the first study across the private hospitals of Iran, it was necessary to use factor analysis for determining the quality dimensions from the patients' perspective. A five-point Likert scale was used, ranging from strongly disagree (1) to strongly agree (5) to assess the level of patients' expectation and perception of service quality.

Data analysis was done using SPSS 17.0 software. Exploratory factor analysis (EFA) was used for determining the dimensions of service quality. Also, the Wilcoxon test was used in comparing the patients' "perception" and "expectations" scores and in analyzing such mean score in the different groups; the *t*-test and Kruskal-Wallis tests were used.

### Ethics

This study was approved by the ethics committee of the Deputy of Research, Tehran University of Medical Sciences (code: 130/1293).

## Results

### Patients' characteristics

Table 1 shows the demographic findings of the patients. The patients' average age was 47.9 years (SD = 16.9). About 898 patients (91%) had insurance coverage. The average length of stay of the patients was 4.6 days (SD = 4.4). A total of 320 patients (33%) had been previously admitted in the current hospital, and 266 patients (27%) had used outpatient services of the current hospital (imaging, laboratory, clinics, and emergency services).

**Table 1:** Socio-demographic data of the sample (N = 983)

Variables		N	%
**Gender**	Male	450	45.8
	Female	533	54.2

**Age**	≤ 30	178	18.1
	31-40	169	17.2
	41-50	228	23.2
	51-60	145	14.8
	≥ 61	263	26.8

**Education level**	Illiterate	64	6.5
	Primary and secondary school	441	44.9
	Academic Degree	478	48.6

**Residence**	Urban	949	96.5
	Rural	34	3.5

**Hospital ward**	Internal	249	25.3
	Surgery	320	32.6
	Obstetrics and Gynecology	168	17.1
	Other	246	25.0

**Health condition**	Excellent	74	7.5
	Good	538	54.7
	Average	331	33.7
	Bad	40	4.1

### Construct validity and reliability

The first aim of the study was to determine the service quality dimensions of private hospitals in Iran from the patients' perspective. The construct validity was determined using EFA (principal components analysis with Varimax rotation method). The sample adequacy for extraction of the factors was confirmed through Kaiser-Meyer-Olkin (KMO) test and Bartlett's test of sphericity. The Bartlett's test result was significant (p < 0.001), and the KMO value (0.975) showed that using exploratory EFA was suitable.

In this analysis, the factors with eigenvalues equal or higher than 1 were considered significant and chosen for interpretation. By EFA, 3 factors were extracted, explaining 69% of the total variance. All factor loadings were higher than 0.4, indicating that they were statistically significant and higher than the recommended level [20]. The factor loading of each item has been listed in Table 2. The EFA results specified three dimensions of the service quality as follows:

**Table 2:** Dimensions of hospital service quality, mean scores for patients' expectations, perceptions, and quality gaps and Wilcoxon test results

Dimensions and items	Factor loading	Mean perception score	Mean expectation score	Mean quality gap scores
**Reliability/Responsiveness**		4.05	4.93	-0.88

1. Sincere interest of personnel in solving patients' problems	0.59	4.04	4.94	-0.89

2. Carrying out of the services right at the first time	0.56	4.08	4.92	-0.84

3. Providing services at appointed time	0.62	4.11	4.92	-0.81

4. Error-free and fast retrieval of documents	0.58	3.97	4.93	-0.96

5. Telling when services will be performed	0.71	4.02	4.90	-0.88

6. Prompt performance of medical and non-medical services	0.74	4.03	4.91	-0.87

7. Willingness of personnel to help patients	0.80	4.08	4.93	-0.84

8. Aattending of personnel whenever called	0.80	4.09	4.94	-0.84

9. Instilling confidence in patients	0.76	4.04	4.95	-0.91

10. Feeling safety and security in interaction with personnel	0.74	4.04	4.95	-0.91

**Empathy**		3.89	4.87	-0.98

11. Polite and friendly dealing of personnel with patients	0.47	4.39	4.96	-0.57

12. Knowledgeable personnel to answer patients' questions	0.59	3.95	4.90	-0.94

13. Individual attention to patients	0.64	3.34	4.87	-1.52

14. Availability of 24-hour services	0.67	3.97	4.94	-0.96

15. Attention to the patient's beliefs and emotions	0.84	3.90	4.86	-0.96

16. Having patients' best interest at heart	0.85	3.83	4.78	-0.94

17. Understanding specific needs of patients	0.82	3.86	4.82	-0.96

**Tangibles**		4.18	4.95	-0.76

18. Neat and well-dressed personnel	0.75	4.36	4.96	-0.59

19. Clean and comfortable environment of the hospital	0.81	4.29	4.97	-0.67

20. Modern and up-to-date equipment	0.69	3.96	4.95	-0.99

21. Visually appeal of physical facilities	0.70	4.12	4.94	-0.81

**Overall Quality**		4.02	4.91	-0.89

• Factor 1 included 10 items relating to the reliability, responsiveness, and assurance, which explained 27.8% of the total variance and was labeled as "reliability/responsiveness."

• Factor 2 includes 5 empathy items and 2 assurance items, which explained 22.1% of the total variance and was named "empathy."

• Factor 3 includes 4 tangible items, which explained 19.1% of total variance and was named "tangibles."

To evaluate the reliability of the three service quality dimensions, the internal consistency analysis was performed. The Cronbach alpha coefficient ranged from 0.85 to 0.95 for perception dimensions and 0.96 for overall perception, as well as 0.80 to 0.90 for expectation dimensions and 0.93 for overall expectation, showing that the instrument is sufficiently reliable.

### Descriptive statistics

Based on our findings, the mean scores of expectations were high and ranged from 4.78 for (item 16: Having patients' best interest at heart) to 4.97 for (item 19: Clean and comfortable environment of the hospital). The total means score of patients' expectation was 4.91. Among the three dimensions, the highest expectation related to the tangibles dimension (dimension's mean score = 4.95) and the lowest expectation related to the empathy dimension (dimension's mean score = 4.87). Among the four items with highest expectation score, three items related to the tangibles and one item related to the empathy dimension. Of the four items with lowest expectation score, all 4 items related to empathy.

The mean score of the perceptions ranged from 3.34 for (item 13: Individual attention to patients) to 4.39 for (item 11: Polite and friendly dealing of personnel with patients). The total means score of patients' perceptions was 4.02. Among the three dimensions of quality, the highest perception related to the tangibles dimension (dimension's mean score = 4.18) and the lowest perception related to the empathy dimension (dimension's mean score = 3.89). Among the four items with highest perception score, 3 items related to the tangibles and one item related to the empathy dimension. From the items with lowest perception score, all 4 items related to empathy. These items had the lowest expectation scores, as well.

The gap score for each item and dimension was computed by subtracting the expectation score from the perception score. The Wilcoxon test results show that the differences between perception and expectation for all the 21 items and 3 dimensions are statically significant (p < 0.001). Also, the difference between the total mean score of perceptions and expectations is statistically significant, and hence, there is a gap between the patients' perception and their expectation of the service quality of Tehran private hospitals (see Table 2).

Our findings show that the highest gap of the quality relates to the empathy dimension (gap mean score = -0.98), and there is a considerable gap between the patients' expectations and perceptions. The lowest gap of the quality relates to the tangibles dimension (gap mean score = -0.76). An overview of 21-items gap scores shows that from those five items with highest gap, four items relate to the empathy dimension (items 13, 14, 15, and 17), and one item relates to the tangibles dimension (item 20), confirming the above-mentioned results (see Table 2).

Investigating the difference between the patients' expectations scores based on the socio-demographic variables showed that there is a relatively significant difference between the expectations mean scores based on gender)t(848) = 2.78, p = 0.05 (and the women's expectations were more than the men's regarding service quality. There was a statistically significant difference between the patients' expectation scores based on the education level (H (2) = 16.64, p = 0.001), and the illiterate patients had higher expectations than the educated ones. Also patients with previous hospitalization in that same hospital had less expectations than the others regarding service quality (t (539) = 2.32, p = 0.02).

Investigating the difference between the patients' perception scores based on the socio-demographic variables regarding quality of the services showed that there is a statistically significant difference between the patients' perception scores among those with and without insurance coverage)t(981) = 2.59, p = 0.01), and patients without insurance coverage had lower quality perception. The difference between the perception scores based on the average length of stay (LOS) was statistically significant (H (4) = 17.88, p = 0.001), and the perceptions score is decreased by the increase in the LOS. The difference between the perceptions' scores based on the patients' health conditions on discharge was statistically significant (H (3) = 18.55, p = 0.001), and patients who had described their health conditions as "excellent" and "good" had higher perception score than others.

## Discussion

The main objective of this study was to provide a conceptual and operational framework to the policy makers and decision makers about the patients' expectations and perceptions of service quality in private hospitals. SERVQUAL questionnaire was used in this study, but results from the factor analysis did not confirm the structure suggested by the Parasuraman et al.[14,18] and three dimensions of reliability, responsiveness, and assurance were converted into a single dimension. In Yasilda and Direjtor study, these three dimensions were converted into one dimension named reliability/confidence [21] and in Dengjuin et al. study; the three dimensions were converted into one dimension named responsiveness [22]. Therefore, the patients in private hospitals of Iran define the quality of services in three dimensions: tangibles, reliability/responsiveness, and empathy.

Generally, the patients have high expectations in private hospitals (4.91 of 5 [≈98%]), which is not unusual and similar to the results of previous studies accomplished in Cyprus [7], Turkey [4] and Taiwan [22,23]. The service quality of private hospitals have been satisfactory from the patients' perspective (4.02 of 5 [≈80%]), although there is much work to do for improvement in all areas of service quality. Similar results have been reported for the quality score in other parts of the world [4,7,22,24].

The highest expectation and perception and lowest gap of quality is related to the tangibles dimension, showing that the private hospitals have paid attention to the physical aspects and infrastructures of care delivery. Our findings confirm two previously carried out study results in Singapore and Malaysia [6,25]. The tangibles dimension entails considerable importance for customer evaluation of service quality [14], so that attractive environment and suitable hotel services are compelling reasons for them to choose a specific private hospital. In recent years, the new private hospitals in Tehran have invested more in physical and environmental besides the medical aspects, considerably satisfying the patients' expectations.

Low perception and expectation score and high gap score of empathy dimension is indicative of a weak relationship between the physician, nurses, and the personnel with patients and need to improve behavior and communication between personnel and patients. This is similar to the results gained from the study by Huang et al [26], but contrary to the results of Jabnoun and Chaker study [27]. The human elements have higher importance relative to nonhuman elements in the patients' perception of the quality of the private health care services [28], and the interpersonal relationships are one of the most important factors in the perception of service quality [29,30]. Results from several studies have shown the importance of the interpersonal relationship component of service quality regarding satisfaction [28,29,31,32] and patient loyalty [2]. The practitioners/personnel must make the patients aware of their disease conditions, answer their questions, recognize and pay attention to their emotional and social needs and be available when needed.

Professional, timely, and proper services are what the patients expect from the hospitals. The quality of services provided by the hospitals is determined mainly by the process-related factors like scheduling, delivery of care in the fastest time, and correctness [31]. Previous study results show that process of care delivery is a determining factor in the patients' perception of the quality of the services, and they are more sensitive to the process of care delivered by the nurses and personnel [4,31,33]. Accordingly, the reliability/responsiveness dimension, focusing on the process of care, still requires more attention to meet the patients' expectations regarding this aspect of quality. Hospitals must design a scheduling system of service provision and be bound to it.

Based on our study results, women's expectation score was higher than that of the men. The women's higher expectations compared with the men had been reported in the previous studies [22,23]. Unlike the results of two studies in Turkey [34] and Taiwan [22] in our study, patients with higher education level had lower expectations than the others. It seems that with higher education levels, the individuals' expectations become more reasonable. The patients with previous admission in the current hospital had lower expectations than the other patients. It seems that their previous experience and recognition has caused them to adjust their expectations in accordance with that specific hospital's facilities and conditions. The patients without insurance coverage had lower perception of quality. Because these patients pay their costs through out of pocket, they expect that the private hospitals meet their expectations. The patients with longer average LOS in the hospital had lower perception of the quality of the services. The relationship between longer average length of stay and lower satisfaction level has been reported in previous studies as well [35]. The patients' health conditions was influential in their satisfaction of the service quality, and those who had described their health conditions as "excellent" or "good" were more satisfied with the provided services. It has been proved in previous studies that better physical and mental health condition has a significant effect on the patients' satisfaction of services [36,37].

Our study also has limitations that restrict the generalizability of the results. First; the results are based on the private hospitals of Tehran city, so other studies must be accomplished in other parts of the country to increase the generalizability of results of this study. Second; the majority of patients were urban and had insurance coverage. Thus, results must be used with cautions.

## Conclusions

The results showed that SERVQUAL is a valid, reliable, and flexible instrument to monitor and measure the services quality in private hospitals of Iran and enables the hospital managers to identify the areas that need improvement from the patients' perspective. The results could be used in the planning for quality improvement by private hospitals. According to the findings, the quality improvement efforts of private hospitals is advised to mostly focus on modernizing equipments, timeliness of care delivery, accuracy of performance as well as on enhancing the interpersonal relationships and communication skills of its physicians, nurses and other personnel.

## Competing interests

The authors declare that they have no competing interests.

## Authors' contributions

AZ- Selected the topic and designed the study, analyzed the data, interpreted the findings, wrote the first draft of the manuscript and revised the manuscript; MA- Selected the topic and designed the study, analyzed the data, interpreted the findings, commented on the first draft of the manuscript and revised the manuscript; ARF- Designed the study, analyzed the data, interpreted the findings and commented on the first draft of the manuscript; AR- Designed the study, analyzed the data, interpreted the findings and commented on the first draft of the manuscript; SMGT- Designed the study, analyzed the data, interpreted the findings and commented on the first draft of the manuscript. All authors read and approved the final manuscript.

## Pre-publication history

The pre-publication history for this paper can be accessed here:

http://www.biomedcentral.com/1472-6963/12/31/prepub
